# Choice of *y*-axis can mislead readers

**DOI:** 10.1007/s00210-020-01926-x

**Published:** 2020-06-25

**Authors:** Betül R. Erdogan, Jan Vollert, Martin C. Michel

**Affiliations:** 1 Department of Pharmacology, Faculty of Pharmacy, Ankara University, Ankara, Turkey; 2 Pain Research, Department of Surgery and Cancer, Imperial College London, London, UK; 3 Department of Pharmacology, Johannes Gutenberg University, Mainz, Germany

**Keywords:** Perception, Bias, Reproducibility, Pre-specification

## Abstract

Using two examples from the non-scientific literature, we show how choice of unit of measure and scaling of *y*-axis can caused a biased perception of data, a phenomenon we propose to call perception bias. We recommend to pre-specify unit of measure or how it will be determined, whether outcome variables will be shown as absolute or relative/normalized changes, and to typically start *y*-axis at 0 for ratio variables.

Reproducibility is essential for reliable, robust, and rigorous scientific research. It has been found that the majority of non-clinical research (up to 89%) in the life sciences, including pharmacology, is not reproducible (Freedman et al. 2015). Low reproducibility rates of non-clinical studies result in waste of money, time, and effort and may be considered inappropriate use of experimental animals. Lack of reproducibility is also a major concern for successful translation of non-clinical into clinical studies. Poor translation of promising treatment into patients has undesirable effects on both human health and the economy (Erdogan and Michel 2020). Several factors including various types of biases such as selection, performance, detection, attrition, and publication bias have been identified to contribute to poor reproducibility in non-clinical research. In recent years, many applicable guidelines emphasize that pre-specification of various elements such as study design, execution, data analysis, and reporting are needed to avoid biases and enhance reproducibility of research (Vollert et al. 2020).

The importance of adequate graphical depiction of data in biomedicine has been emphasized before (Franzblau and Chung 2012) but not linked to reproducibility. It was highlighted outside of biomedicine, for instance in chemistry (Szafir 2018), sustainability research (Cüre et al. 2020) and accounting (Burgess et al. 2008), how frequently used types of graphical representation may mislead readers. However, this aspect has gained little attention in current guidelines related to reproducibility (Vollert et al. 2020). Using two examples from public media and outside of life sciences, we show how unit of measure and scaling of *y*-axis can cause biased perception of results, a phenomenon we propose to call perception bias.

Figure 1 shows the results from a 2019 state election in Germany. All 3 depictions of the election outcome are factually correct, and all three have been shown on federal, public service media. However, depending on unit of measure of the *y*-axis, each panel creates a very different perception on which party has “won”: while the red party got the greatest share of votes (Fig. 1a); compared with the election 5 years ago, the brown party gained most percentage points (Fig. 1b), and the yellow party had the largest % increase (Fig. 1c). Unit of measure is a common issue in biomedicine, very often as part of normalization. For instance, protein expression data are often shown after normalization for expression of a reference gene product. This can be appropriate if expression of the reference gene product is stable but can become misleading if it is not. Thus, treatment of human embryonic kidney cells stably transfected with β_3_-adrenoceptors with the agonist isoprenaline reduced the abundance of the G-protein α-subunits of G_i1_ and G_s_ and did not change that of G_i2_ (Michel-Reher and Michel 2013). Concomitantly such treatment reduced the abundance of GAPDH by about 30% (Michel-Reher and Michel 2015), which is frequently use to normalize data derived from immunoblots. Had such normalization been applied to the G-protein expression data, the reductions in G_i1_ and G_s_ would have disappeared, and the lack of change of G_i2_ turned into a decrease, i.e., a very different conclusion reached. A second example is the normalization of contraction data from organ bath experiments based on size of the tissue strips (Erdogan et al. 2020). Comparing contraction of urinary bladder strips from young and old rats, a reduced response to the agonist carbachol had been observed if contraction had been normalized to strip weight, but no such difference was found when it had been normalized to strip length (Schneider et al. 2005). A third and final example comes from the diabetes field. While animal models of type 1 diabetes typically have a reduced body weight, those of type 2 diabetes in most cases have an increased body weight; accordingly, the urinary bladder appears reduced or unchanged when normalized for body weight, but unchanged or increased, respectively, when not adjusted for body weight (Ellenbroek et al. 2018). Since the denominator body weight differentially affects type 1 and 2 diabetes, the decision to use this denominator biases findings from type 1 models towards finding greater and those from type 2 models towards finding smaller enlargement.

**Fig. 1:**
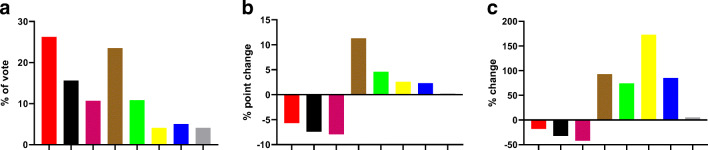
Results of election in the German state of Brandenburg held on 1.9.2019. **a** Share of vote. **b** Percentage point change of votes in comparison with 2014 values. **c** % change of votes in comparison with 2014 values (Statistisches Bundesamt 2019). (Figures were generated using GraphPad Prism, version 8.3)

Figure 2 is an example from a leading German daily newspaper, Die Welt. It shows reduction in share of cars with a diesel engine among newly registered cars in Germany. The graph as published in the newspaper (left panel) uses a *y*-axis starting at 40%, and the regression line creates the perception that cars with a diesel engine will cease to exist soon (needless to say this did not happen). If the same data are plotted using a neutral *y*-axis scale starting at 0 (right panel), the declining trend remains clear, but the decline is much less steep, and there is no perception that diesel engines will cease to exist in the foreseeable future. A variation of this theme is the choice of aspect ratio, i.e., whether a graph is wide and short or tall and skinny, which can create distinct impressions. Getting back to the specific example shown here, the extrapolation of the correlation line beyond the measured data (as provided in the original publication) further creates a perception that would only be justified if past events can be extrapolated into the distant future, which is generally accepted to be untrue.

**Fig. 2:**
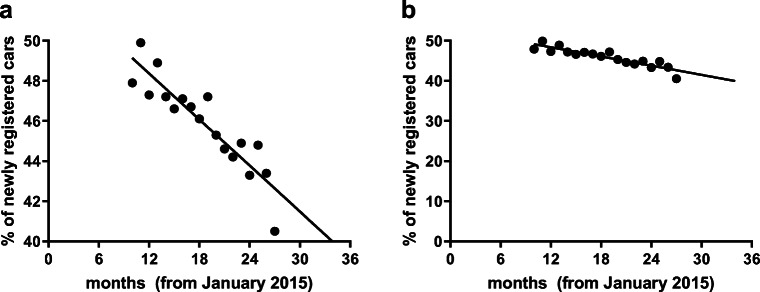
Percent of newly registered cars powered by a diesel engine in Germany. **a** Redrawn based on the originally published graph (i.e., identical scaling of *y*-axis) (Anonymous 2017). **b** Redrawn using *y*-axis starting at 0. (Figures were generated using GraphPad Prism, version 8.3)

These simple examples highlight how easily unit of measure and scaling of *y*-axis affect our perception about the results—even for scientists being used to accessing them on a daily basis. Thus, presentation of data has an impact on what the findings convey to readers/reviewers and can cause misleading impressions about outcomes of the study. Both real life examples demonstrate that choice of unit of measure (Fig. 1) and/or scaling of *y*-axis (Fig. 2) can cause bias in the perception and interpretation of the data. In analogy to other type of biases which are well known to effect reproducibility of non-clinical studies, we propose to call this phenomenon perception bias.

Based on these considerations, we propose the following:

- As with other types of bias, pre-specification of what and how to show experimental findings in the study protocol is an essential protection against bias in the graphical representation of data. However, we realize that this is not always feasible, particularly in exploratory studies. If pre-specification is not feasible, a second-best option is to specify the rules how unit of measure and scaling of y-axis will be determined. If neither is feasible, authors should carefully consider whether their choice of graphical representation may unduly nudge readers to one of several interpretations of the data.
- Axis scaling should cover the full range of the data. The scale should be biologically or clinically meaningful. The default should be to start at 0 for all ratio variables; while exceptions from this rule can be very adequate (for instance when displaying mammalian body temperature, heart rate, or blood pressure), it is upon authors to explain why axis scaling does not start at 0. For instance, it has been argued that specific *y*-axis scaling may be appropriate to avoid overlapping in graphics (In and Lee 2017). It should be avoided to emphasize group differences by a short *y*-axis if the observed differences relative to normal variability of a parameter are small.
- Absolute changes describe something different than relative changes (see Fig. 1). Depending on the scientific question at hand, either can be justified but the choice should not be driven by the desirability of the outcome. When in doubt, both should be presented.
- Finally, within a given article authors should be as consistent as possible in the way they graphically represent the data.

We hope that a more conscientious approach to choice of unit of measure and scale of axes will lead to more transparency in reporting and thereby help to improve reproducibility.

## References

[CR1] Anonymous (2017) Trend spricht gegen Diesel. Die Welt 6.4.2017, Axel Springer SE, Berlin

[CR2] Burgess DO, Dilla WN, Steinbart PJ, Shank TM (2008). Does graph design matter to CPAs and financial statement readers?. J Bus Econ Res.

[CR3] Cüre T, Esen E, Calikan AÖ (2020). Impression management in graphical representation of economic, social and environmental isses: an empirical study. Sustainability.

[CR4] Ellenbroek JH, Arioglu Inan E, Michel MC (2018). A systematic review of urinary bladder hypertrophy in experimental diabetes: Part 2. Comparison of animal models and functional consequences. Neurourol Urodyn.

[CR5] Erdogan BR, Michel MC (2020) Building robustness into translational research. In: Bespalov A, Michel MC, Steckler T (Eds) Good Research Practice in Non-Clinical Pharmacology and Biomedicine, Handbook of Experimental Pharmacolology, Vol. 257, p. 163, 175. Springer Verlag, Heidelberg 10.1007/164_2019_283 31598837

[CR6] Erdogan BR, Karaomerlioglu I, Yesilyurt ZE, Oztürk N, Muderrisoglu AE, Michel MC, Arioglu Inan E (2020) Normalization of organ bath contraction data for tissue specimen size: does one approach fit all? Naunyn Schmiedeberg's Arch Pharmacol 393:243–251. 10.1007/s00210-019-01727-x 10.1007/s00210-019-01727-x 31511953

[CR7] Franzblau LE, Chung KC (2012). Graphs, tables, and figures in scientific publications: the good, the bad, and how not to be the latter. J Hand Surg [Am].

[CR8] Freedman LP, Cockburn IM, Simcoe TS (2015) The economics of reproducibility in preclinical research. PLoS Biol 13:e1002165. https://doi.org/10.1371%2Fjournal.pbio.1002165 10.1371/journal.pbio.1002165 PMC4461318 26057340

[CR9] In J, Lee S (2017) Statistical data presentation. Kor J Anesthesiol 70:267–276. 10.4097/kjae.2017.70.3.267 10.4097/kjae.2017.70.3.267 PMC5453888 28580077

[CR10] Michel-Reher MB, Michel MC (2013). Agonist-induced desensitization of human ß_3_-adrenoceptors expressed in human embryonic kidney cells. Naunyn Schmiedeberg's Arch Pharmacol.

[CR11] Michel-Reher MB, Michel MC (2015). Regulation of GAPDH expression by treatment with the ß-adrenoceptor agonist isoprenaline - is GAPDH a suitable loading control in immunoblot experiments?. Naunyn Schmiedeberg's Arch Pharmacol.

[CR12] Schneider T, Hein P, Michel-Reher M, Michel MC (2005). Effects of ageing on muscarinic receptor subtypes and function in rat urinary bladder. Naunyn Schmiedeberg's Arch Pharmacol.

[CR13] Statistisches Bundesamt (2019) Amtliches Ergebnis der Landtagswahl in Brandenburg am 1. September 2019. https://de.statista.com/statistik/daten/studie/37811/umfrage/endergebnis-der-landtagswahl-in-brandenburg-im-jahr-2009/. Accessed 23 May 2020

[CR14] Szafir DA (2018). The good, the bad, and the biased. Five ways visulaization can mislead (and how to fix them). Interactions.

[CR15] Vollert J, Schenker E, Macleod M, Bespalov A, Wuerbel H, Michel M, Dirnagl U, Potschka H, Waldron A-M, Wever K (2020) Systematic review of guidelines for internal validity in the design, conduct and analysis of preclinical biomedical experiments involving laboratory animals. BMJ Open Science 4:e100046. https://doi.org/10.1136/bmjos-2019-100046 10.1136/bmjos-2019-100046 PMC8647591 35047688

